# NUF2 Drives Cholangiocarcinoma Progression and Migration via Inhibiting Autophagic Degradation of TFR1

**DOI:** 10.7150/ijbs.80737

**Published:** 2023-02-21

**Authors:** Jijun Shan, Wangjie Jiang, Jiang Chang, Tao Zhou, Yananlan Chen, Yaodong Zhang, Jifei Wang, Yuming Wang, Yirui Wang, Xiao Xu, Shuochen Liu, Xiaoli Shi, Shilong Fan, Ruixiang Chen, Changxian Li, Xiangcheng Li

**Affiliations:** Hepatobiliary Center, The First Affiliated Hospital of Nanjing Medical University

**Keywords:** Cholangiocarcinoma, NUF2, TFR1, MAPK, Autophagy

## Abstract

Cholangiocarcinoma (CCA) is the second most common primary hepatic malignancy and associated with poor prognosis. Lack of therapeutic methods for CCA and insensitivity of targeted therapy and immunotherapy make its treatment challenging. NUF2, a component of Ndc80 kinetochore complex, is implicated in the initiation and development of multiple cancers. However, the role and mechanism of NUF2 in CCA is still unclear. In this research, we investigated the biological processes and underlying mechanisms of NUF2 in CCA. We discovered that the expression of NUF2 was upregulated in CCA and negatively correlated with prognosis. Changes in NUF2 levels had an impact on cell proliferation and migration. Moreover, NUF2 functioned as an oncogene to promote the progression of CCA through p38/MAPK signaling by inhibiting p62 binding of TFR1 and affecting its autophagic degradation. In addition, TFR1 promoted CCA progression and Kaplan-Meier analyses uncovered patients with high expression of TFR1 was associated with the poor survival. In conclusion, our study demonstrated that NUF2 promoted CCA progression by regulating TFR1 protein degradation, and the NUF2/TFR1/MAPK axis could be an excellent therapeutic target for CCA.

## Introduction

Cholangiocarcinoma (CCA) is a heterogeneous group of malignancies arising from varying locations within the biliary tree, which is associated with poor prognosis 1. CCA is the second most frequent type of primary liver cancer, accounting for approximately 15% of all primary liver tumors and 3% of all gastrointestinal cancers 2. Although in some countries CCA is a rare cancer, the morbidity and mortality are increasing exceptionally worldwide 3, 4. The most common classification of CCA is intrahepatic cholangiocarcinoma (iCCA), perihilar cholangiocarcinoma (pCCA), and distant cholangiocarcinoma (dCCA) according to anatomical location, which also have different oncologic characters, clinical features, treatment strategies, and prognosis 5-7. At present, a cure may only be achieved by radical resection or liver transplant after strict selection of early-stage disease; however, most patients present with advanced stage and lost the opportunity of surgery because of no obvious symptoms at the early stage 8-10. Due to the scarcity of research on biomarkers and drug targets, CCA therapy is in a slow process. Hence, more effective biomarkers are urgent to be found for diagnosis and treatment 11-13.

The correct chromosome segregation during mitosis depends on the interaction between the spindle microtubules and the kinetochore, and it is well-recognized that aberrant chromosomal separation during mitosis is a common cause of carcinogenesis 14, 15. NUF2, the component of Ndc80 kinetochore complex, is a crucial substance that maintains spindle microtubule-kinetochore connection throughout the metaphase of cell division 16, 17. Previous studies showed that depletion of NUF2 resulted in a strong prometaphase block with an active spindle checkpoint 18. Therefore, it is not surprising that the change of NUF2 expression leading to its dysfunction can promote tumor development. This has been verified in renal clear cell carcinoma 19, colorectal and gastric cancers 20. However, there has been no research on the connection between NUF2 and CCA. So little is understood about the precise role, clinical implications, and underlying mechanism of NUF2 in CCA.

TFR1, the transferrin receptor, is an essential protein involved in the uptake of iron and regulation of cell growth 21. Iron is necessary for DNA synthesis as well as other biological functions. Malignant cells frequently overexpress TFR1 due to its essential role in the pathophysiology of cancer cells, and this increased expression has been linked to a poor prognosis 22. TFR1 is an appealing target for antibody-mediated therapy due to the high levels of TFR1 expression on malignant cells, as well as its extracellular accessibility, capacity for internalization, and crucial role in the pathophysiology of cancer cells. Although TFR1 has been used as a popular target for antibody-mediated cancer therapy over the years, interest in both targeting the receptor for delivery and using it as a direct anticancer agent is growing 23.

Autophagy is a conserved catabolic system that destroys extra or malfunctioning cell components through the lysosome 24. It was reported that perturbations in autophagy have been found in many cancers, which have dual functions of promoting tumor and inhibiting tumor 25. Autophagy is a lysosomal-mediated process including autophagosomes formation, autophagosome-lysosomal fusion, and autophagolysosomal degradation 26. Autophagy consists of over 30 core autophagy-related proteins, including LC3B and p62. Double membrane vesicles are considered as a typical feature of autophagy. These vesicles could engulf the damaged proteins and organelles and fuse with lysosomal vesicles.

This study aimed to explore the functions and mechanisms of NUF2 in CCA. Our results demonstrated that high expression of NUF2 was closely related to poor prognosis, and NUF2 can act as an independent predictor for CCA. In addition, our *in vitro* experiments emphasized the role of NUF2/TFR1/MAPK axis in the progression of CCA, and can be used as a new target for the treatment of CCA in the future.

## Materials and Methods

### Cell culture and transfection

The human normal bile duct cell line HIBEpiC and cholangiocarcinoma cell lines, QBC939, RBE, HuCCT1 and HCCC9810 used in this study were purchased from the Chinese National Human Genome Center (Shanghai, China). Cells were cultured in DMEM culture medium supplemented with 10% fetal bovine serum (FBS)and 1% penicillin/streptomycin under 5% CO_2_ at 37 °C. NUF2 and TFR1 knockdown were used by siRNA. Cells were transfected with 50 nM siRNA against NUF2 (5'-GUGGCAUUAUCAACUUUAUTT-3', 5'-GCUACAACAAUCACUAAAUTT-3', 5'-CACCAAGAAUGAUCUUUAUTT-3'), TFR1 (5'-UAGAGAAUGCUGAUCUAGCUU-3', 5'-AACTTCAAGGTTTCTGCCAGC-3'), p62 (CUUCCGAAUCUACAUUAAATT) or scrambled control siRNA (Genepharma Co., Ltd, Shanghai, China) using lipofectamine 2000. The NUF2 overexpression lentiviruses and TFR1 overexpression plasmid were constructed by Cories Biotechnology Co., Ltd. (Nanjing, China). The cells were transfected according to the manufacturer's protocol. Knockdown and overexpression efficiency was evaluated by western blot.

### Human Tissue Samples and Tissue Microarray (TMA)

A cohort of 38 pairs of CCA tissues and adjacent normal tissues were obtained from CCA patients who underwent curative resection in The First Affiliated Hospital of Nanjing Medicine University. Outdo Biotech Company (Shanghai, China) made the tissue microarray using samples from CCA patients who underwent surgery at The First Affiliated Hospital of Nanjing Medicine University between 2006 and 2017. The expression of NUF2 and TFR1 was scored by two independent pathologists and all differences that arose were resolved by discussion. The intensity was classified as negative (0), weak (1), moderate (2), or strong (3), and the density of positive cells in the target region was scored as follows: 0-25% (1), 26-50% (2), 51-75% (3), and 76-100% (4). The overall score was calculated by multiplying intensity and density. The expression level was categorized as low (0-4) and high (6-12). We showed representative IHC images for the scoring criteria in Figure S1. All the patients understood the procedure and provided written consent for the study.

### Transcriptomic RNA-sequencing (RNA-seq)

Three pairs of QBC939/siNC and QBC939/siNUF2 were prepared for RNA-seq analysis by Personalbio (Shanghai, China). The fold change cut-off identified a significant difference in mRNA expression (P-value < 0.05 and |log2 FC| >1) between groups.

### Co-immunoprecipitation and mass spectrometry

Cells were lysed with Nonidet P-40 Substitute. Protein complexes were captured using specific antibodies and IgG at 4 °C overnight and protein A+G magnetic beads were used to capture the antigen antibody complex the next day. Mixed at room temperature for two hours, discarded the supernatant, and obtained the antigen antibody complex for subsequent experiments. The protein complexes were separated by SDS-PAGE and detected with corresponding antibodies by western blot assays. Cut out candidate targets with obvious differences from the gel and send them to the company for mass spectrometry for protein identification.

### mRFP-GFP-LC3 puncta assay

Cells were plated in a 24-well plate with appropriate density and transfected with mRFP-GFP-LC3 adenovirus (Genepharma Co., Ltd, Shanghai, China) for 72h. Then we used a confocal microscope (Zeiss, LSM880) to observe cells. mRFP was utilized to label and track LC3. GFP expression was reduced, indicating that lysosomes and autophagosomes had combined to create autophagolysosomes, and GFP was quenched as a result of a pH adjustment, at which time only red fluorescence was seen. Following microscopic imaging, the red and green fluorescence pictures were combined; the yellow and red puncta were regarded as autophagosomes and autophagolysosomes, respectively. The quantity of yellow and red puncta was counted to determine the intensity of autophagy flux.

### Transmission electron microscopy (TEM)

Autophagosomes were observed by transmission electron microscopy. 2.5% glutaraldehyde was used to chemically fix cells for 30 minutes at room temperature. The cells were then put into pyramid-tip moulds, dehydrated using a graduated series of ethanol, and polymerized for 72 hours at 60 °C. Sections that were cut to a thickness of 100 nm were gathered. The grids were stained for 20 minutes with 1% uranyl acetate, then left at room temperature for 10 minutes with Reynolds lead citrate. A Hitachi H-7650 transmission electron microscope (Hitachi High-Tech, Japan) operating at 80 kV was used to analyze the sections. Software called AMT-600 Image Capture Engine was used to take the pictures.

### Immunofluorescence assay

Fixed cells by 4% paraformaldehyde at 37 °C for 45 min and permeabilized by 0.3% Triton X-100 for 15 minutes. Then block cells with 5% donkey serum for 30 min and incubated them with specific primary antibodies at 4 °C overnight. We developed fluorescence by incubating with Cy3, 488 or FITC labeled goat anti-rabbit IgG for 2 h at room temperature. The nucleus was stained with DAPI. Fluorescence image were captured by a confocal fluorescence microscope.

### Statistical Analysis

All experiments were repeated at least three times. The results were presented as the mean ± standard error of the mean, unless stated otherwise. SPSS v24.0 (IBM, SPSS, Chicago, IL, USA) and Graphpad Prism 9 (GraphPad Software, La Jolla, CA, USA) were used to perform statistical analyses. Differences between the two groups were analyzed by Student's t-test. Moreover, Overall survival and disease-free survival were analyzed with Kaplan-Meier methods, and log-rank test was applied for comparison. A multivariate analysis was performed using Cox proportional hazard regression model. A p-value of <0.05 was considered significant: *p < 0.05, **p < 0.01, and ***p < 0.001.

More materials and methods were recorded in supplementary file.

## Results

### NUF2 was upregulated in CCA and correlated with poor prognosis

**Figure 1:**
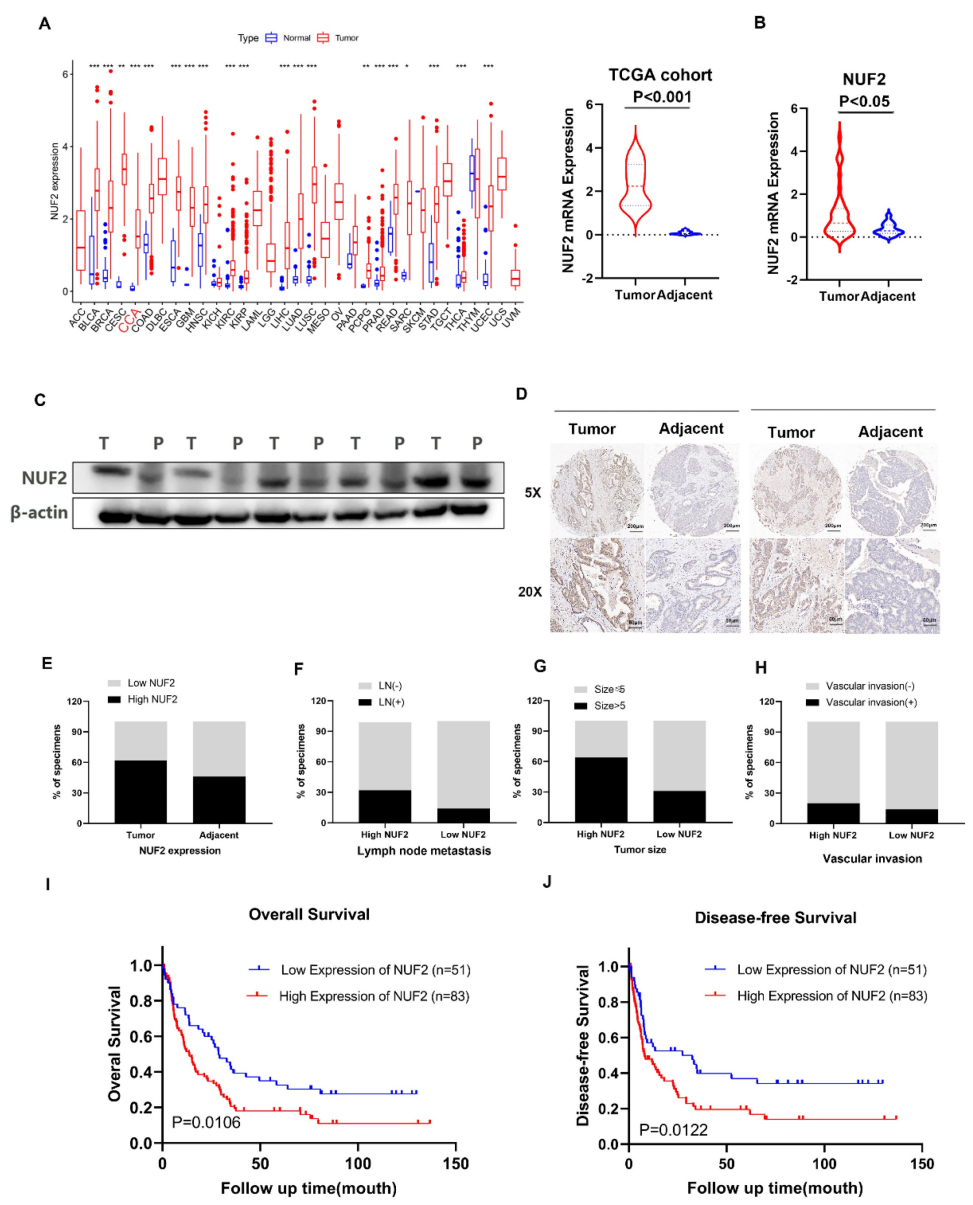
NUF2 was up regulated in CCA and predicated the poor prognosis. (A) Pan-cancer analysis of NUF2 and analysis of NUF2 mRNA level in CCA tissues and adjacent tissues using TCGA database. (B) qRT-PCR analysis of NUF2 expression in CCA tissues and adjacent tissues. (C) Western blot analysis of NUF2 expression in CCA tissues and adjacent tissues. (D) Immunohistochemical staining images of NUF2 in CCA tissues and adjacent tissues (Scale bar: 200 μm, 50 μm). (E) Statistical analysis of NUF2 expression according to the IHC assay. (F-H) Ratio of lymphatic metastasis, tumor size>5cm, and vascular invasion in high NUF2 group and low NUF2 group. (I) Overall survival and (J) Disease-free survival related to NUF2 expression by Kaplan-Meier survival curve analysis.

Our previous RNA-seq found that NUF2 was upregulated in CCA tissues. The pan-cancer analysis of NUF2 in TCGA database showed that NUF2 was highly expressed in most tumors, including in CCA (Figure 1A). We next examined 38 pairs of CCA tissues in our center, and found that the mRNA expression of NUF2 was upregulated in CCA tissues compared to matched normal tissue samples (Figure 1B). Western blot also showed that the protein level of NUF2 was significantly increased in tumor tissues (Figure 1C). To analyze the relationship between NUF2 expression and prognosis, we performed tissue microarrays by IHC staining in 134 CCA patients. The results showed that NUF2 expression was higher in tumor tissues than matched normal bile duct tissues (Figure 1D-E). The ratio of lymphatic metastasis, tumor size>5cm, and vascular invasion was significantly higher in NUF2 high expression group than that in low expression group (Figure 1F-H). Kaplan-Meier survival analysis showed that patients with high expression of NUF2 had a shorter overall survival (OS) and disease-free survival (DFS) compared with low- NUF2 CCA patients (Figure 1I-J). These results indicated that NUF2 might be a critical regulator in CCA progression and functioned as an independent predictive factor of prognosis for CCA patients.

### NUF2 promoted CCA cell proliferation and migration *in vitro*

We then explored the role of NUF2 in CCA progression *in vitro* by evaluating cell proliferation and migration. Compared with normal biliary epithelial cells, the expression of NUF2 was significantly higher in human CCA cell lines QBC939, RBE and HCCC-9810 (Figure S2A). We next chose QBC939 with the highest expression to knock down, and HuCCT1 with the lowest expression to overexpress. The transfected efficiency was validated by western blot (Figure S2B).

**Figure 2:**
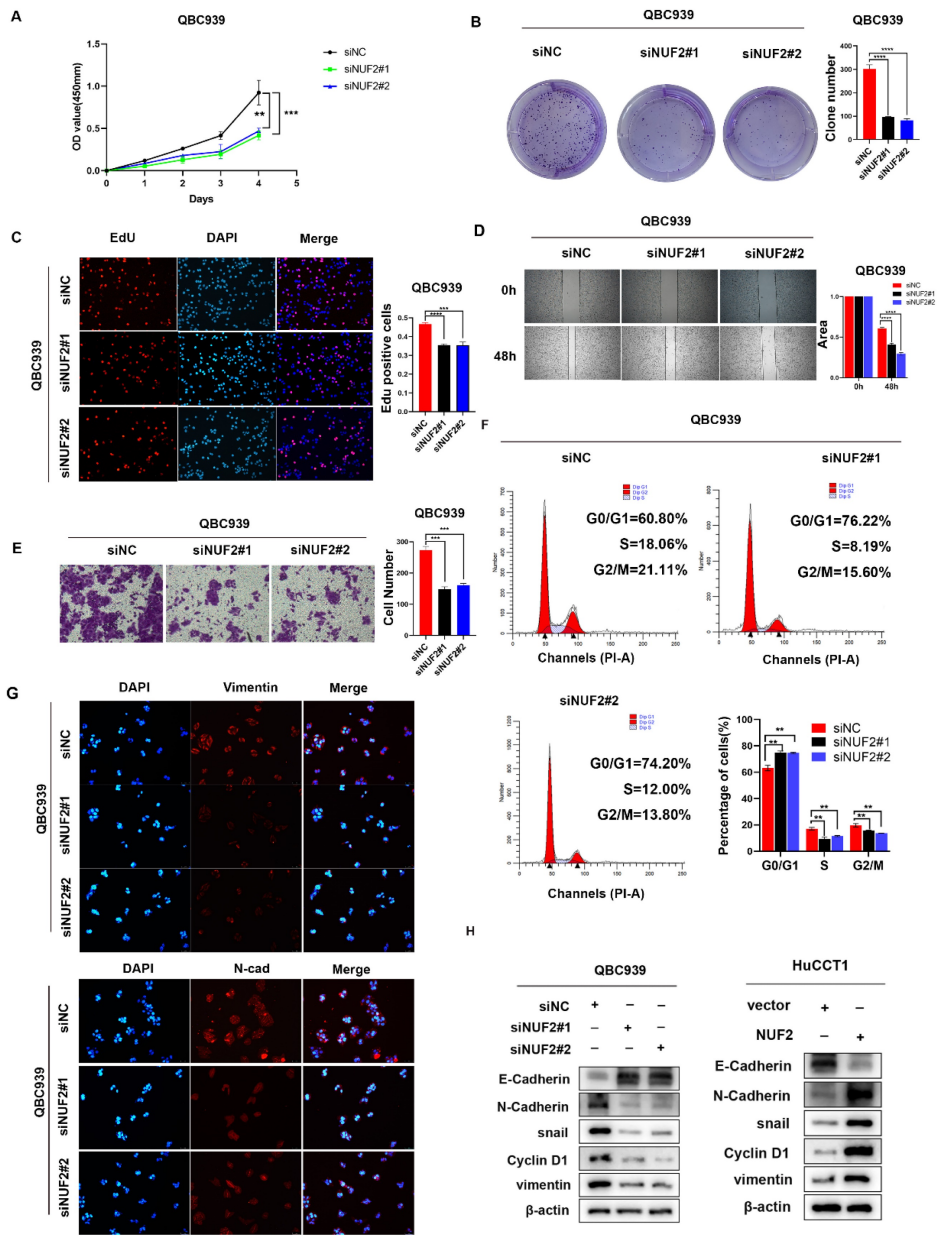
NUF2 promoted CCA cell proliferation and migration *in vitro*. (A) CCK8 assays, (B) clone formation, (C) EdU assays were performed to identify the proliferation ability after NUF2 knockdown or overexpression. (D) Wound healing and (E) transwell assays were performed to identify the migration ability after NUF2 knockdown or overexpression. (F) Flow cytometry analysis of the cell cycle of CCA cells. (G) The expression of N-cad and Vimentin was evaluated by IF staining. (H) Western blot analysis of the levels of vimentin, N-cadherin, E-cadherin, and snail in HuCCT1 (upper) and QBC939 (lower) cells.

CCK8 assays results showed that knocking down NUF2 significantly inhibited the cells proliferation of CCA, while overexpression of NUF2 promoted cells proliferation (Figure 2A and Figure S3A). The clone formation assays demonstrated that NUF2 knockdown weakened the ability of clone formation, whilst NUF2 overexpression had the opposite situation (Figure 2B and Figure S3B). With EdU assays, the percentage of red cells which means that the cells in the proliferation phase decreased significantly after knocking down NUF2, while overexpression of NUF2 significantly increased the percentage of red cells (Figure 2C and Figure S3C). Furthermore, the results of wound healing and transwell assays showed that depletion of NUF2 notably attenuated the migration ability of QBC939 cells while overexpression of NUF2 significantly promoted the migration ability of HuCCT1 (Figure 2D-E and Figure S3D-E). Flow cytometry analysis showed that NUF2 knockdown significantly increased the proportion of cells in the G0/G1 phase and decreased the proportion of cells in the S and G2/M phase. And the percentage of cells in the S and G2/M phase was significantly higher in the NUF2 overexpression group, while the percentage of cells in theG0/G1 phase decreased significantly (Figure 2F and Figure S3F). Moreover, we found that knockdown of NUF2 promoted the expression of E-cadherin and inhibited the expression of N-cadherin, snail, vimentin (Figure 2G-H and Figure S3G). Overexpression of NUF2 had the opposite phenomenon. All these results indicated that NUF2 played an essential role in the cell proliferation, migration, and EMT of CCA.

### NUF2 promoted CCA progression via the MAPK signaling pathway

**Figure 3:**
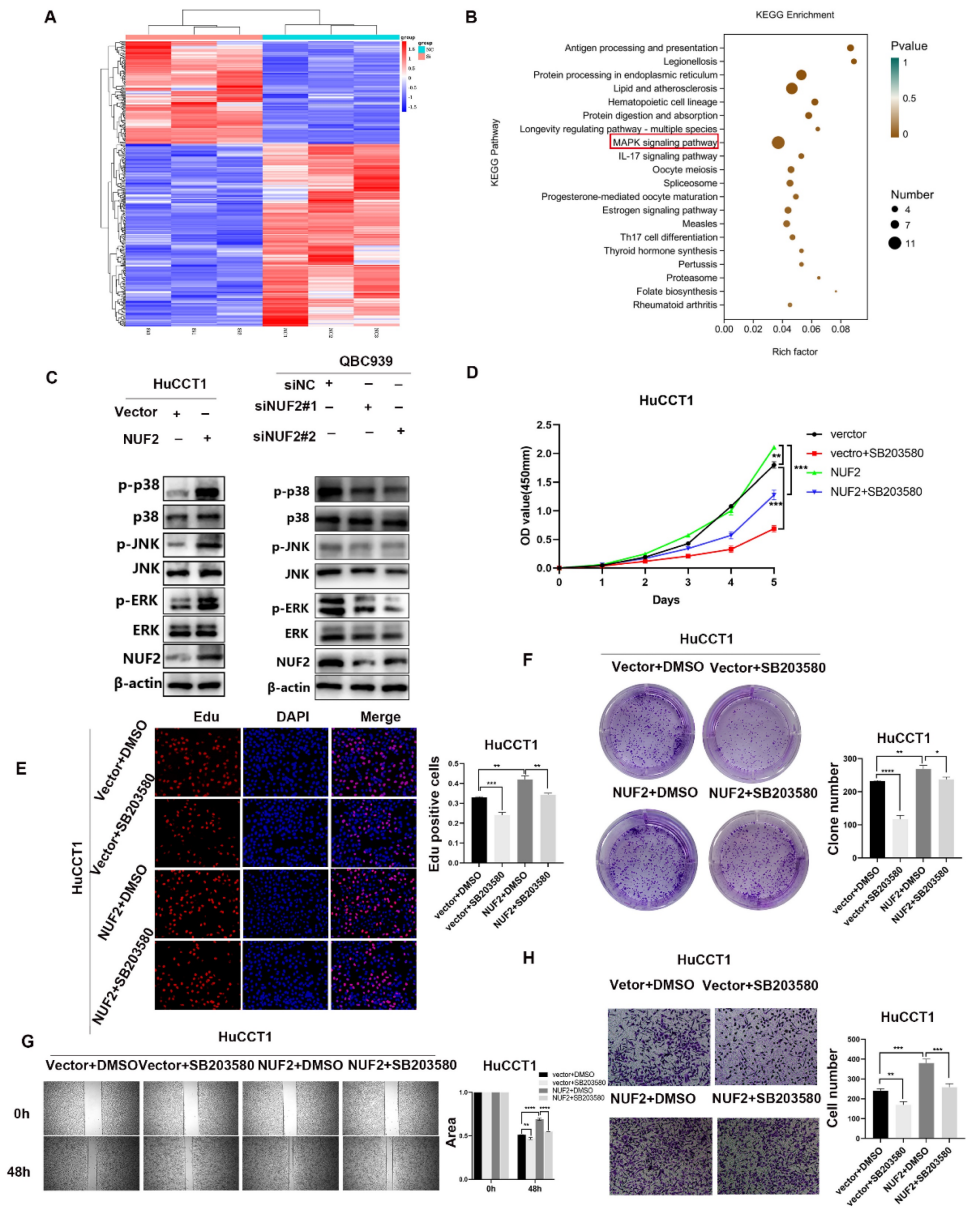
NUF2 promoted CCA cell proliferation and migration via the p38/MAPK signaling pathway. (A) Hot map of 361 differential genes in the RNA-seq. (B) Top 20 signaling pathway maps enriched in the RNA Sequence. (C) Western blot analysis of NUF2, p-JNK, p-p38, p-ERK, JNK, p38, and ERK protein levels. (D) CCK8 assays, (E) EdU assays, and (F) clone formation showed SB203580 reverses the promotional effect of NUF2 overexpression on the proliferation of CCA cells. (G) Wound healing and (H) Transwell assays showed SB203580 reverses the promotional effect of NUF2 overexpression on the migration of CCA cells.

To unlock the mechanism how NUF2 regulated CCA progression, we compared the transcriptome profiles of QBC939 cells with NUF2 knockdown and controls by RNA-seq analysis. A total of 361 differential genes were found, including 130 upregulated genes and 231 downregulated genes (Figure 3A). In KEGG analysis, the top 20 signaling pathways according to the p-value were represented in Figure 3B. The MAPK pathway contained the most differential genes and was the only one closely related to cell proliferation and migration in the RNA-seq result, suggesting that this pathway may be important in NUF2 regulating CCA progression. Further research was done to determine which of the three main MAPK signaling subgroups was responsible for the carcinogenic function of NUF2. Compared to control cells, all three subgroups were downregulated in NUF2 knockdown cells, especially in p-p38 and p-ERK (Figure 3C). Overexpression of NUF2 promoted the activations of p-p38 and p-ERK.

To understand the importance of the p-p38 or p-ERK in NUF2-mediated CCA progression, we performed rescue experiments using the p38 inhibitor SB203580 and ERK inhibitor U0126 (Figure S4). Regretfully, U0126 could not inhibit the tumor-promoting effect caused by NUF2 (Figure S5). However, SB203580 restored the CCA cells proliferation and migration caused by NUF2 overexpression. The CCK8, EdU assays and clone formation demonstrated that compared with the DMSO groups, the addition of p38 inhibitor significantly reduced the CCA cells proliferation (Figure 3D-F). Wound healing and transwell assays showed that the migration ability was weakened after adding p38 inhibitor (Figure 3G-H). These results implied that NUF2 promoted CCA progression maybe through regulating MAPK signaling pathway.

### NUF2 interacted and co-localized with TFR1

**Figure 4:**
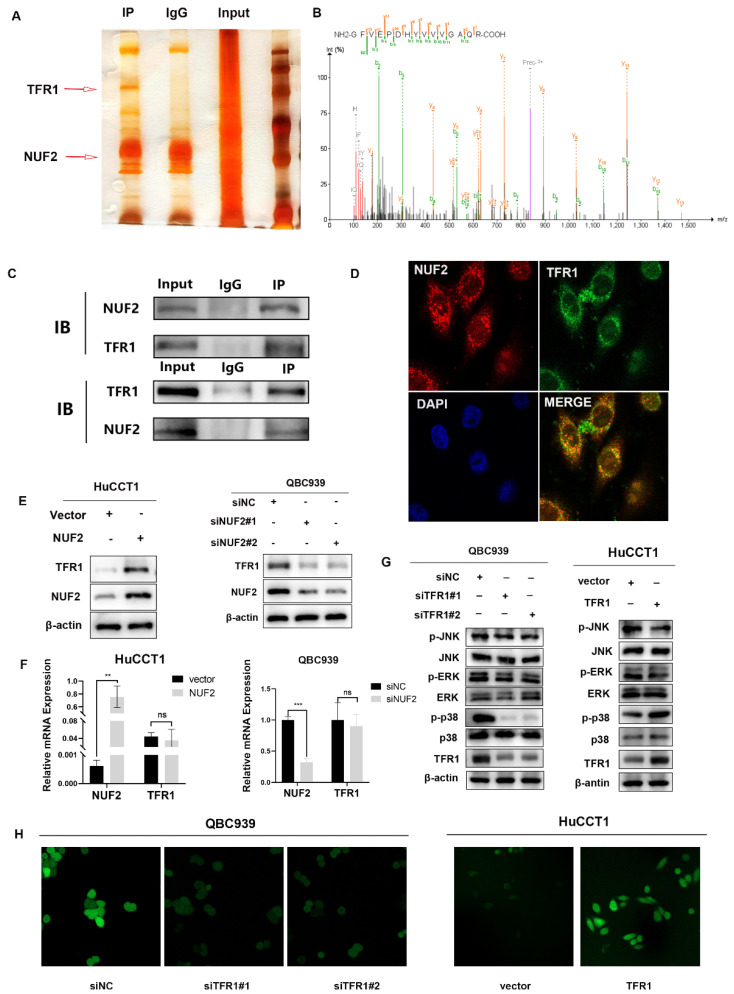
NUF2 interacted and co-localized with TFR1. (A) NUF2-associated proteins analyzed by MS. (B) Mass spectrogram of TFR1. (C) Co-IP analysis of NUF2 and TFR1. (D) Confocal immunofluorescence analysis of NUF2 and TFR1 expression. (E) Changes of TFR1 protein level after NUF2 knockdown or overexpression. (F) Changes of TFR1 mRNA level after NUF2 knockdown or overexpression. (G) Western blot analysis of TFR1, p-JNK, p-p38, p-ERK, JNK, p38, and ERK protein levels. (H) Intracellular ROS levels were evaluated.

To further identify the potential mechanism of NUF2 regulating MAPK, a combined IP/MS approach was used to identify novel NUF2-mediated protein- protein interactions (Figure 4A). We found that TFR1 had the highest score among the differential proteins closely related to MAPK pathway in comparison of the CO-IP products by NUF2 antibody and IgG (Figure 4B). To confirm the MS data, we performed the CO-IP assay to verify the binding between two proteins (Figure 4C). Furthermore, confocal immunofluorescence microscopy captured a high degree of spatial concordance between endogenous NUF2 and TFR1 in QBC939 cells (Figure 4D). These findings demonstrated that NUF2 could interact with TFR1. Then we investigated whether the change of NUF2 protein expression could synchronously cause the change of TFR1 expression. The results showed that the protein level of TFR1 was upregulated in NUF2 overexpression group, while was down- regulated in NUF2 knockout group (Figure 4E). However, there was no obvious change on the mRNA level (Figure 4F). We then used public database to verify our experiments and the public data showed that NUF2 had no correlation with TFR1 at mRNA level (Figure S6). Furthermore, we also verified the changes of three MAPK subgroups after TFR1 knockdown or overexpression. We found that the difference of p-p38 in three subgroups was the most obvious, and no significant change in p-ERK and p-JNK (Figure 4G). Chirasani et al. showed that TFR1 can activate p38/MAPK pathway, thereby promote tumor progression by increasing intracellular ROS concentration 27. In our results, overexpression NUF2 promoted accumulation of intracellular ROS, while knocking down NUF2 decreased the level of intracellular ROS (Figure 4H). These results indicated that NUF2-TFR1-p38 axis maybe play a major role in regulating CCA progression.

### TFR1 promoted CCA cell proliferation and migration

**Figure 5:**
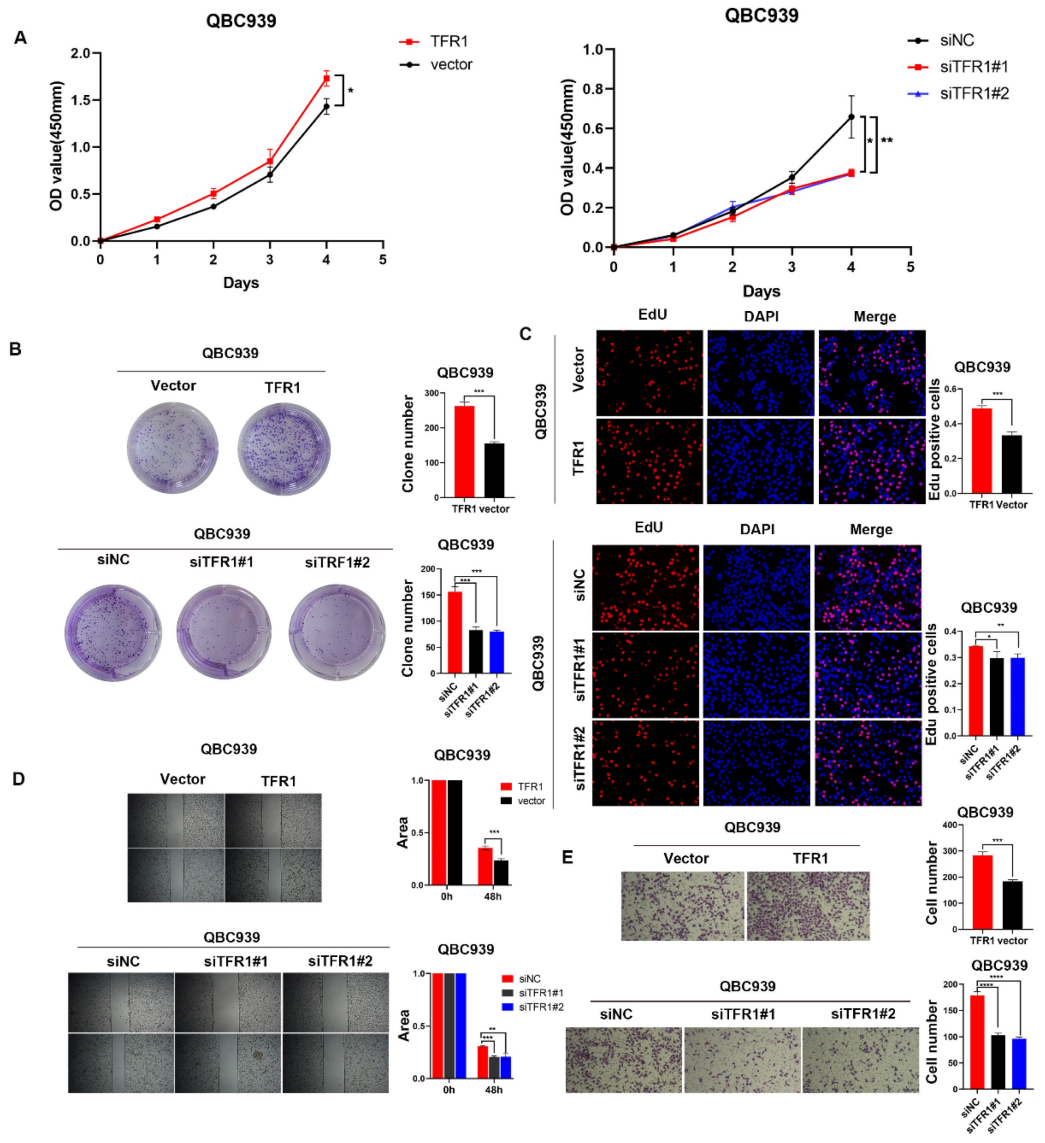
TFR1 promoted CCA cell proliferation and migration *in vitro*. (A) CCK8 assays, (B) clone formation, and (C) EdU assays were performed to identify the proliferation ability. (D) Wound healing and (E) transwell assays were performed to identify the migration ability.

To explored the function of TFR1 in CCA, we knocked down and over expressed TFR1 in CCA cells. The efficiency was examined by western blot (Figure S7). We used CCK8, clone formation, EdU assays, wound healing and transwell assays to explore the capacity of proliferation and migration. The results showed that TFR1 knockdown impaired the CCA cells' capacity for proliferation and migration, while TFR1 overexpressing promoted the CCA cells proliferation and migration (Figure 5A-E).

### TFR1 was correlated with poor prognosis

**Figure 6:**
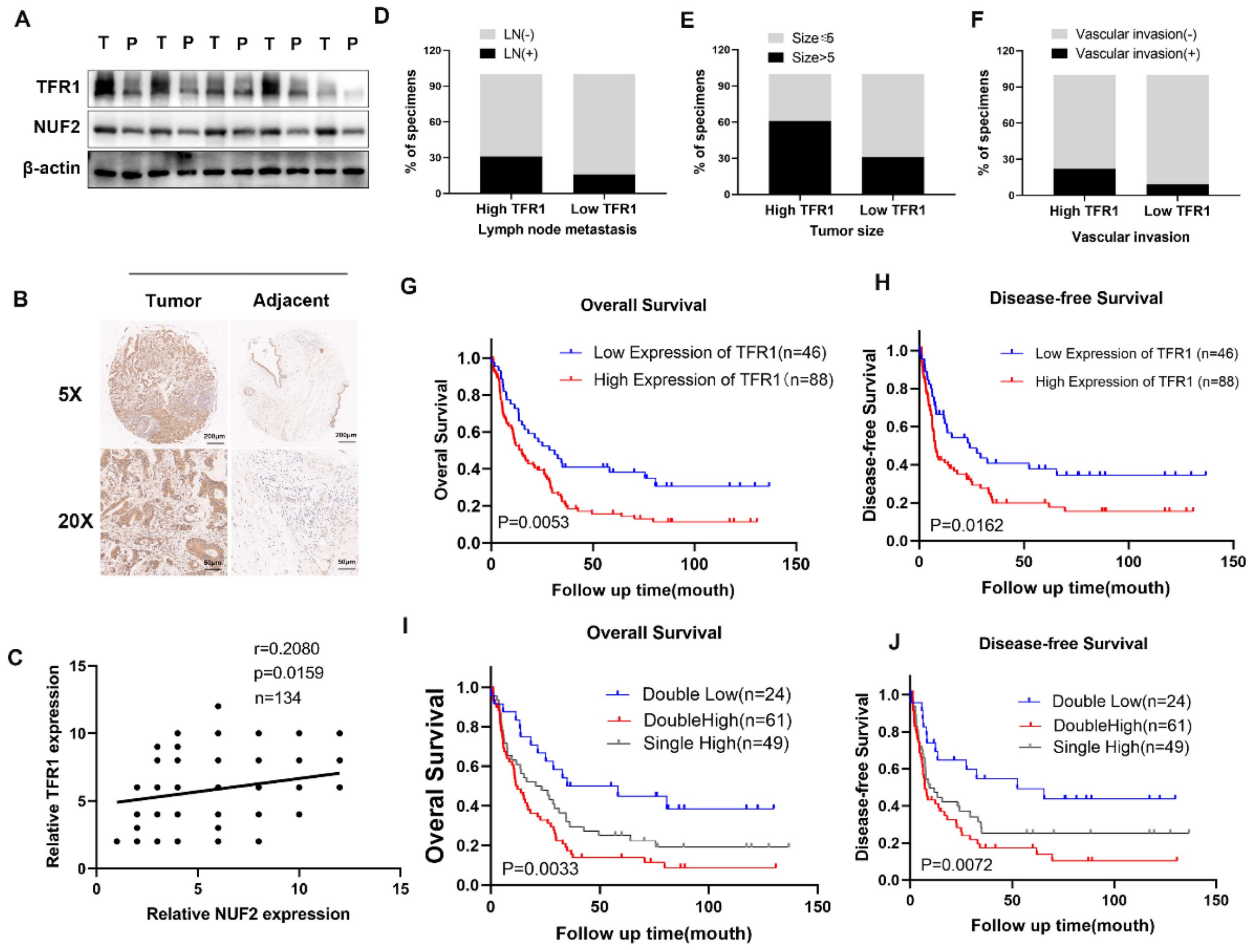
Clinical significance of TFR1. (A) Western blot analysis of NUF2 and TFR1expression in CCA tissues and adjacent tissues. (B) Immunohistochemical staining images of TFR1 in CCA tissues and adjacent tissues. (C) Correlation analysis of NUF2 and TFR1 expression according to IHC staining. (D-F) Ratio of lymphatic metastasis, tumor size>5cm, and vascular invasion in high TFR1 group and low TFR1 group. (G) Overall survival and (H) Disease-free survival related to TFR1 expression by Kaplan-Meier survival curve analysis. (I) Overall survival and (J) Disease-free survival were further analyzed by dividing patients into subgroups with the double high expression, single high expression, and dual low expression of NUF2 and TFR1.

Western blot showed that the protein level of NUF2 and TFR1 was significantly increased in tumor tissues (Figure 6A). Immunohistochemical staining of CCA tissues showed that TFR1 was upregulated in CCA tissues (Figure 6B) and it was positively correlated with NUF2 expression (r = 0.2080, p < 0.05) (Figure 6C). In addition, high expression of TFR1 was associated with lymph node metastasis, tumor size, and vascular invasion. People with high TFR1 expression had worse performance in these clinical indicators (Figure 6D-F). Kaplan-Meier analysis showed that CCA patients with high expression of TFR1 had shorter overall survival and disease-free survival (Figure 6G-H). The prognosis of people with both high expression of NUF2 and TFR1 was the worst, and people with both low expressions had the best prognosis (Figure 6I-J). These results indicated that TFR1 might be a critical regulator in CCA progression.

### NUF2 regulated TFR1 degradation through p62-dependent autolysosome pathway

**Figure 7:**
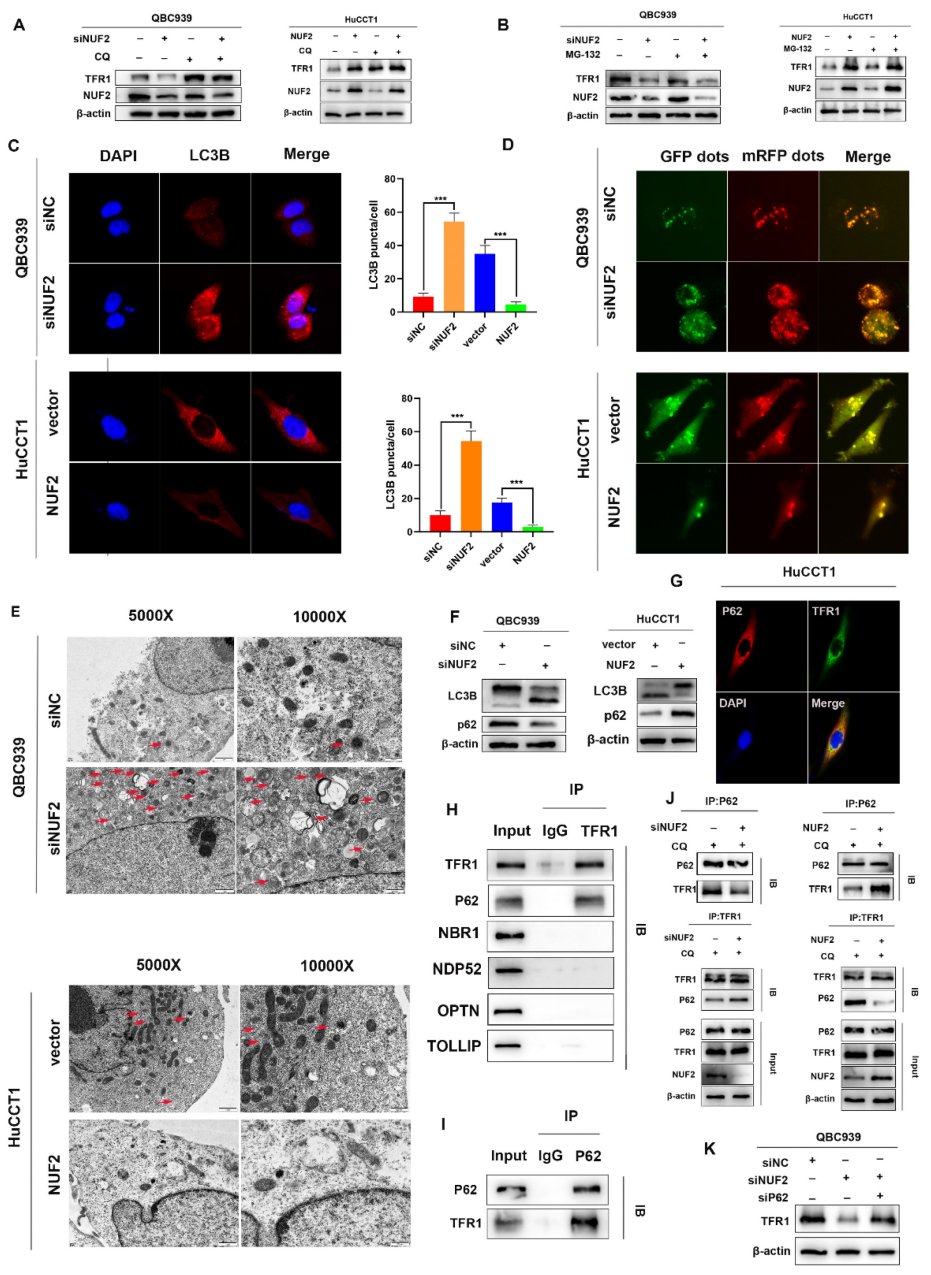
Knockdown of NUF2 promoted autophagy. (A) Cells with NUF2 knockdown or overexpression were treated with CQ (50μM). (B) Cells with NUF2 knockdown or overexpression were treated with MG-132 (10μM). (C) Immunofluorescence confirmed the levels of LC3B involved in autophagy NUF2 knockdown or overexpression in HuCCT1 (upper) and QBC939 (lower) cells. (D) mCherry-GFP-LC3 analysis showed Autophagic flux in CCA cells. (E) Typical transmission electron microscopic images of endogenic autophagic double membrane structures in CCA cells. (F) The protein levels of LC3B and p62 were detected by western blot analysis. (G) Confocal immunofluorescence analysis of TFR1 and p62 expression. (H) IP analysis of the binding of TFR1 and cargo receptors. (I) IP analysis of p62 and TFR1. (J) CO-IP demonstrated the combining ability of TFR1 and p62 in NUF2 knockdown or overexpression cells. (K) Western blot showed that si-p62 can rescue TFR1 protein level in NUF2 knockdown cells.

Next, we further explored how NUF2 regulated TFR1 protein expression. As we all know, there are two main ways of protein degradation: the ubiquitination-proteasome degradation pathway and lysosomal degradation pathway 28. MG132 and chloroquine (CQ) are two mainstream inhibitors, which inhibit ubiquitination-mediated degradation and autophagy-regulated degradation respectively. Hence, in order to find out which way played the major role, QBC939 and HuCCT1cells were treated with MG132 or CQ. And we found that CQ restored the expression of TFR1 protein but not MG132 (Figure 7A-B). The results suggested that NUF2 regulated TFR1 degradation maybe through the autolysosome pathway.

To prove our hypothesis, we examined the expression of LC3B by confocal fluorescence microscopy. The results showed that knockdown of NUF2 induced the accumulation of LC3B while overexpression of NUF2 decreased the expression of LC3B (Figure 7C). Then after GFP-mRFP-LC3 lentivirus transfected in QBC939 and HuCCT1 cells, there were more yellow puncta which indicated an increased autophagic flux in siNUF2 group compared to siNC group while a significant decrease when overexpressing NUF2 (Figure 7D). Furthermore, we found that NUF2 knockdown cells contain abundant autophagic vacuoles while NUF2 overexpression was reversely viewed by transmission electron microscopy (TEM) (Figure 7E). We next measured the protein expression level of LC3B and p62 which are the most important autophagy-related markers. NUF2 overexpression potently downregulated LC3B protein level but increased p62 protein level in HuCCT1 while NUF2 knockdown reversed the situation (Figure 7F). These results implied NUF2 inhibited the autophagic flux in CCA cells.

Substantial pieces of evidence suggested that cargo receptors are essential for delivering substrates to the autophagosome for selective degradation 29. Although p62 is the most well-known autophagy cargo receptor in autophagy, there are still some other cargo proteins that act as transporters. Next, we carried out a series of IP experiments on common autophagy cargo receptors to find out which was truly combined with TFR1. The results demonstrated that only p62 and TFR1 had obvious binding rather than other cargo receptors NBR1, NDP52, TOLLIP and OPTN (Figure 7H-I), and immunofluorescence experiment also verified the binding (Figure 7G). Next, we hypothesized that NUF2 affected autophagy by regulating TFR1 and NUF2 binding. We transfected the siNUF2 and siNC into QBC939 cells, added with autophagy inhibitor CQ as well as. Then TFR1 antibody were used for IP. As a result, we found that NUF2 knockdown group combined with more p62 in cells treated with CQ and overexpression of NUF2 led to consistent conclusions (Figure 7J). Importantly, si-p62 could rescue TFR1 protein level in NUF2 knockdown CCA cells (Figure 7K). These results indicated that NUF2 inhibited autophagy contributed to the accumulation of TFR1 in a p62-dependent manner.

### NUF2 promoted CCA progression via TFR1/p38 signaling pathway

**Figure 8:**
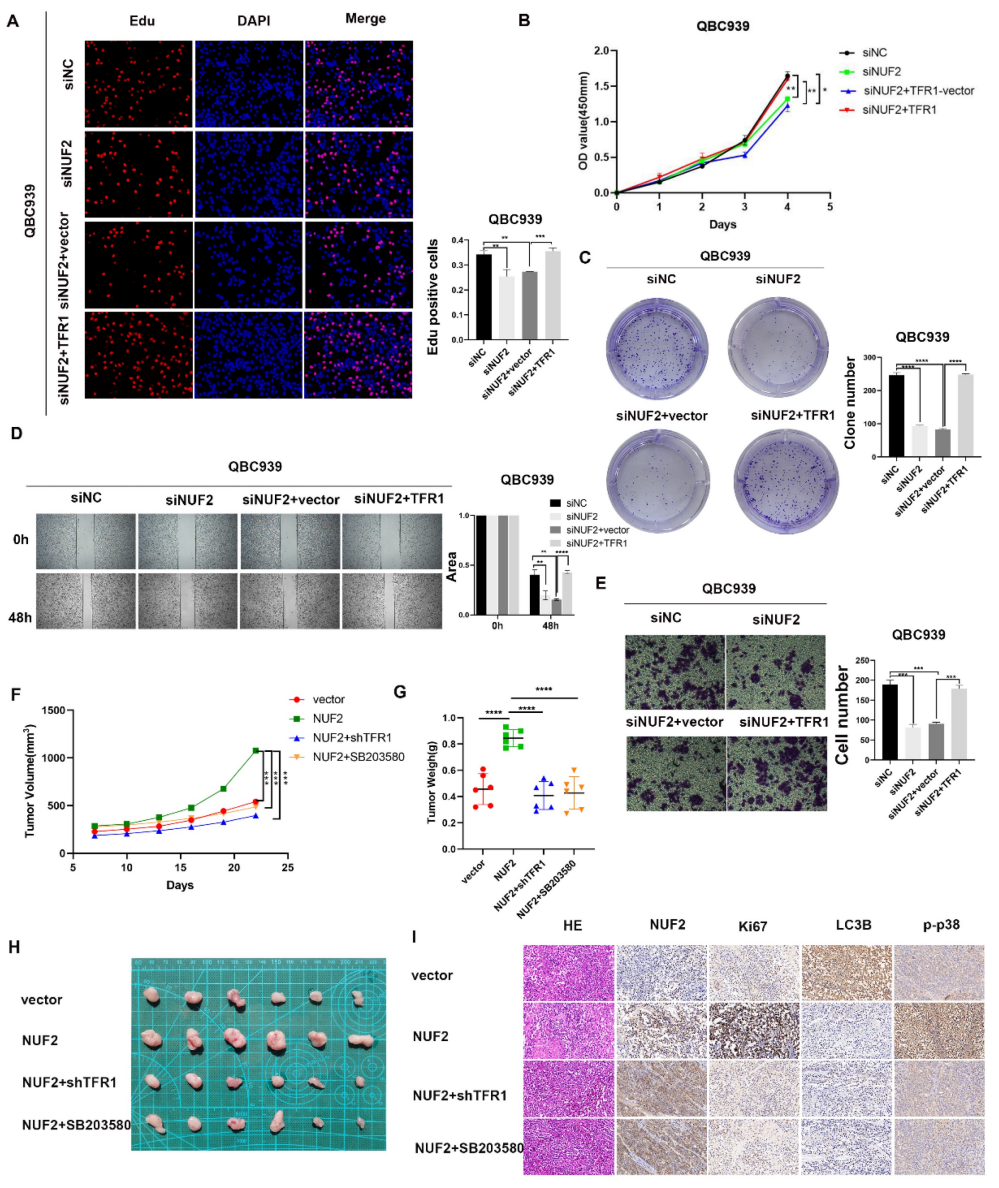
TFR1 reversed the effect of NUF2 on CCA cells. (A) EdU assays, (B) CCK8 assays, and (C) Clone formation showed TFR1 overexpression reversed the inhibited effect of NUF2 knockdown on the proliferation of CCA cells. (D) Wound healing and (E) transwell assays showed TFR1 overexpression reversed the inhibited effect of NUF2 knockdown on the migration of CCA cells. (F-G) Volume and weight of xenograft tumor (n = 6). (H) Typical images of subcutaneous xenograft tumor. (I) Ki67, NUF2, LC3B, and p-p38 expression in different groups of xenografts.

Next, we continued to verified the role of TFR1 in NUF2 regulating CCA progression. The clone formation, CCK8 and EdU assays were used to explore the proliferation ability of CCA cells (Figure 8A-C). Wound healing and transwell assays were performed to detect the effect on cell migration (Figure 8D-E). As expected, overexpression of TFR1 significantly reversed the tumor inhibitory effect of NUF2 knockdown on cell proliferation and migration, which suggested that TFR1 was involved in NUF2 induced CCA malignancy, at least partly through the regulation of TFR1. In addition, a xenograft model was used to investigate the role of NUF2/TRF1/p38 pathway in CCA *in vivo*. The result showed that overexpression of NUF2 promoted the tumor growth. However, the inhibition of TRF1 and p38 impaired the CCA growth caused by NUF2 overexpression (Figure 8F-H). In addition, IHC staining of xenografts showed that tissues from NUF2 overexpression group showed an increase Ki67 expression (Figure 8I). These results implied that the NUF2-TFR1-p38 axis played important roles in CCA progression.

## Discussion

NUF2 is an oncogene that reported in clear cell renal cell carcinoma 30, melanoma 31, hepatocellular carcinoma 32, and breast cancer 33. More importantly, in a phase I clinical trial, NUF2 peptide vaccination has been used to treat castration resistant prostate cancer (CRPC), and achieved encouraging results 34. Together, these findings showed that NUF2 played a unique function in the development and growth of tumors, making it a viable and efficient target for molecule-targeted therapy in a variety of malignancies. Regretfully, there was no study on the role of NUF2 in CCA. In our research, genomics and proteomics data implied that the mRNA and protein abundances of NUF2 were high in the tumor tissues of CCA, and patients with higher NUF2 expression had a poorer prognosis. Univariate and multivariate Cox regression analyses revealed that NUF2 was an independent predictive factor for the prognosis of CCA patients and this was consistent with the results of others' researches 32, 35, 36. Further results showed that NUF2 promoted the proliferation and migration of CCA cells *in vitro*, as well as *in vivo*.

Up to now, most studies have shown that NUF2 mainly affected tumorigenesis by regulating cell cycle and DNA replication 36, 37, which was consistent with the function of NUF2 itself. Nevertheless, the direct targets that mediated NUF2's biological actions in malignancies, particularly in CCA, were not well understood. In this study, we performed RNAseq and mass spectrometry analysis and identified TFR1 and MAPK which served as cancer promoting factors to promote the development of various tumors 38-42. Therefore, we first explored the role of NUF2/TFR1/MAPK axis in CCA. Our rescue experiments proved that NUF2 played an important role in the proliferation and migration of CCA cells through TFR1/MAPK. Our results showed that NUF2 could interact with TFR1, and control the downstream pathway by changing its protein expression. Compared with the control group, after NUF2 knockdown, TFR1 protein expression decreased, cell proliferation and migration ability weakened. Mechanistically, NUF2 inhibited the autophagic degradation of TFR1 by preventing the binding of TFR1 and p62, leading to the accumulation of TFR1 in cells and promoting the occurrence and development of tumors. As a result, our research advanced knowledge of the precise molecular processes underpinning NUF2-mediated CCA development.

TFR1 is a type II transmembrane glycoprotein which regulates iron import and is highly expressed in proliferating exuberantly cells, such as tumor cells 40, 43, 44. TFR1 is abnormally expressed in many human cancers. As a cancer promoting protein, it was reported to induce cancer cell proliferation by protecting cancer cells against natural killer (NK) cells 45 and inhibiting apoptosis 46, as well as regulating NF-KB 47, MAPK 48, AKT 49, and JAK/STAT pathways 50. These results explain why NUF2 could promote the proliferation and migration of CCA via TFR1 in our study. In addition, TFR1 functioned as a universal prognosis predictor 51. Increased expression of TFR1 correlated with advanced stage and poorer prognosis in multiple cancers 52, 53. It was reported that TFR1 could be degraded by ubiquitin proteasome 54 or ubiquitinated and degraded in lysosome 55, 56. According to our experiments, we found that TFR1 was mainly degraded by autophagic lysosome pathway in CCA. The autophagy-lysosomal system is crucial for intracellular protein degradation, nutrition cycling, scavenging, and stability maintenance 57. Although TFR1 has been reported to be degraded by autophagy, the specific mechanism was still unknown. We studied for the first time how NUF2 played a role in the autophagic degradation of TFR1 through p62, and uncovered the molecular mechanism of NUF2 promoting the progression of cholangiocarcinoma through TFR1.

Overactivation of MAPK signaling is a common event in multiple cancers. MAPK pathway contains three major subtypes, including ERK, JNK, and p38. Among them, p38 kinase plays a key role in the occurrence and development of CCA by promoting proliferation, invasion, inflammation and angiogenesis 58, 59. In our research, we found that NUF2 activated p38/MAPK by increasing TFR1 protein expression. ERK and JNK did not seem to play an obvious role in the TFR1/MAPK pathway. This was consistent with other's work 60. As a transmembrane glycoprotein involved in Iron transport, the essential roles of TFR1 in various biological events, such as oxygen delivery, electron transport, and enzymatic reactions, depend on its redox activity 61-63. TFR1 can promoted mitochondrial respiration and ROS production, both of which are key players in tumor cell growth and survival 64. In addition, p-p38 is a major ROS target and ROS can affect expression of p-p38 in cholangiocarcinoma 65. It has been reported that TFR1 can activate p38/MAPK pathway, thereby promote tumor progression by increasing intracellular ROS concentration 27.

In general, we found a new effect of NUF2 on the proliferation and metastasis of CCA cells, revealing how NUF2-TFR1 interaction impacted the stability of TFR1 to activate p38/MAPK signaling, which can work as an ideal therapeutic target in future. Consequently, targeting NUF2 or NUF2-TFR1 interaction may provide a promising therapeutic approach for CCA.

## Supplementary Material

Supplementary methods, figures and table.

## Figures and Tables

**Figure 9:**
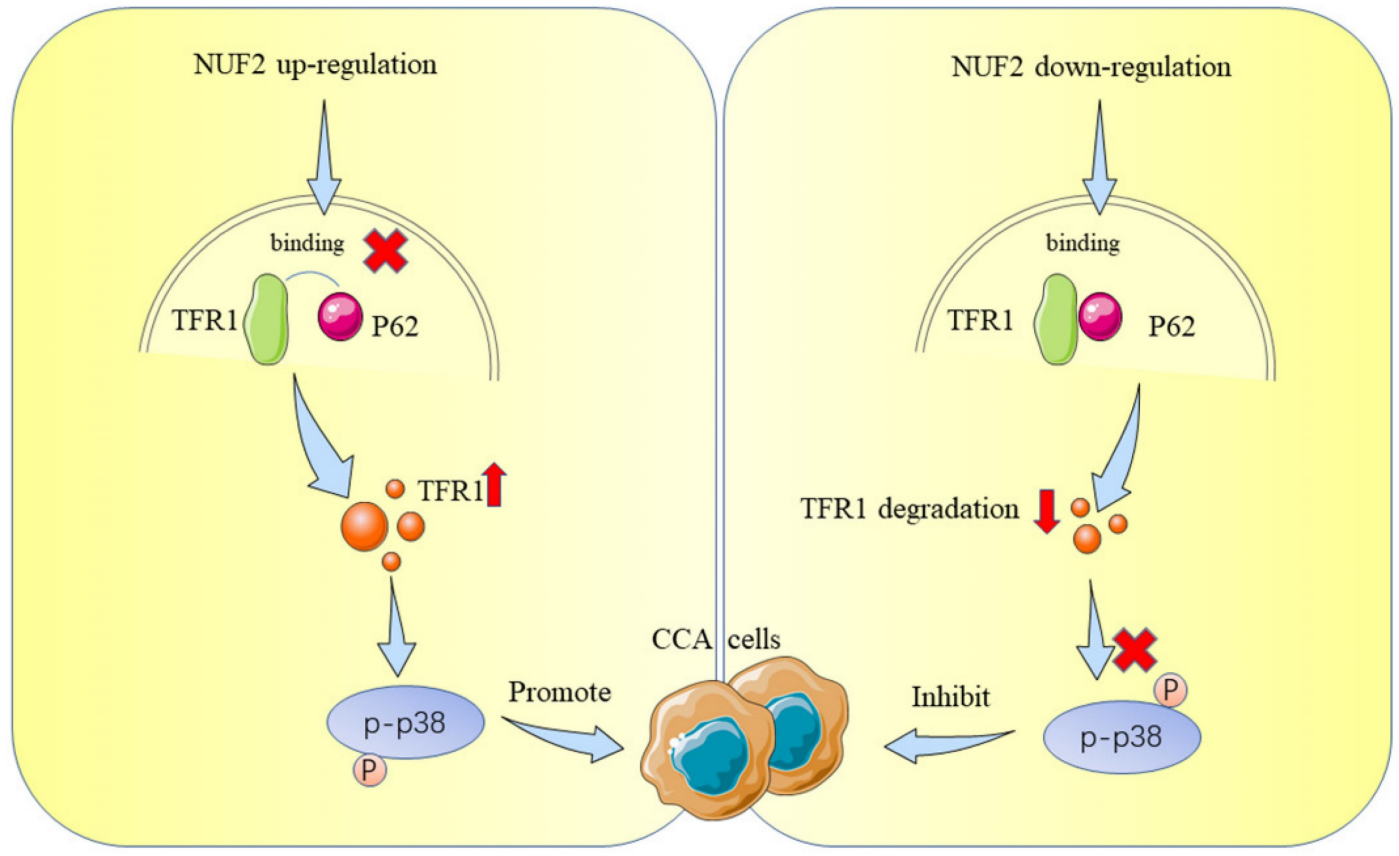
Schematic representation showing the proposed mechanism of NUF2.

## Abbreviations

CCA: cholangiocarcinoma
HCC: hepatocellular carcinoma
iCCA: intrahepatic cholangiocarcinoma
pCCA: perihilar cholangiocarcinoma
dCCA: distant cholangiocarcinoma
TMA: tissue microarray
(RT)PCR: real-time reverse-transcription
siRNA: small interfering RNA
shRNA: short hairpin RNA
IP: immunoprecipitation
TEM: transmission electron microscopy
IF: Immunofluorescence
EMT: epithelial mesenchymal transformation
TCGA: The Cancer Genome Atlas
MAPK: mitogen activated protein kinase
IHC: immunochemistry staining
