# Supplementary material for: NUF2 Drives Cholangiocarcinoma Progression and Migration via Inhibiting Autophagic Degradation of TFR1
Source: Int J Biol Sci. 2023 Feb 21;19(5):1336–51. doi: 10.7150/ijbs.80737 (PMC10086752; doi:10.7150/ijbs.80737)
Supplement: Supplementary file 1 — Supplementary methods, figures and table. [file ijbsv19p1336s1.pdf]

# NUF2 Drives Cholangiocarcinoma Progression and Migration via Inhibiting Autophagic Degradation of TFR1

Jijun Shan[1#], Wangjie Jiang[1#], Jiang Chang[1#], Tao Zhou[1#], Yananlan Chen[1], Yaodong Zhang[1], Jifei Wang[1], Yuming Wang[1], Yirui Wang[1], Xiao Xu[1], Shuochen Liu[1], Xiaoli Shi[1], Shilong Fan[1], Ruixiang Chen[1], Changxian Li[1*], Xiangcheng Li[1*]

List

1. Detailed experimental methods
2. Supplementary table
3. Supplementary figure

**Detailed experimental methods：**

RNA extraction and real-time reverse-transcription (RT) PCR

Total RNA was extracted by TRIzol reagent (Life Technologies). Reverse transcription of total RNA to obtain cDNA by using the RT-PCR kit (Promega). Then the cDNA was amplified with specific primers using a SYBR Green PCR Kit (Qiagen). The following primers were used: NUF2, 5′-GGAAGGCTTCTTACCATTCAGC-3′ (forward) and 5′-GACTTGTCCGTTTTGCTTTTGG-3′ (reverse); TFR1, 5′-ACCATTGTCATATACCCGGTTCA-3′ (forward) and 5′-CAATAGCCCAAGTAGCCAATCAT-3′ (reverse); and β-actin, 5′-CATGTACGTTGCTATCCAGGC-3′ (forward) and 5′-CTCCTTAATGTCACGCACGAT′ (reverse).

Western blotting

Split the total protein with RIPA lysis buffer, centrifugated it at 12000g for 15 minutes, took the supernatant, added loading buffer at 4:1, heat it at 100 °C for 10 minutes, and obtained the protein for Western. The proteins were electrophoresed on 10–15% SDS polyacrylamide gels and then transferred to PVDF membranes. The membranes were

placed in quick blot liquid for 40 minutes at room temperature and incubated with antibodies overnight at 4 °C. After washing with TBST three times, the membranes were incubated with secondary antibodies. Target proteins were detected by using an enhanced ECL kit. Antibodies used in the research were listed in supplementary table 1.

Wound healing assay

The cells of the experimental group and the control group were respectively seeded into 6-well plates. After complete attachment, a 200μ pipette tip was used to make straight lines. The images were recorded with a microscope at the beginning (0h) and end (48h) of the experimental time. The wound closure was calculated according to the following formula: (1- [current wound size/initial wound size]) × 100.

Transwell assay

About $2 \times 10^4$ cells were seeded into the upper chamber with 300 μ medium without FBS. Then we put chambers in 24well plates containing medium with 700 μ medium with 20% FBS at the bottom. After incubation at 37 °C for 48 h, the cells on the upper side migrated to the lower surface of the chamber and then we fixed the chamber with 4% paraformaldehyde and then stained with 0.1% crystal violet solution. Three independent experiments were analyzed.

CCK8 assay

One thousand cells were seeded in a well of a 96-well plate with 100 μl medium. Waiting complete attachment, cells were incubated in 100 μl reaction mixture (10 μl CCK-8 and 90 μl DMEM) for 2 h and measured at a wavelength of 450 nm. The measurements were performed at the 0h, 24h, 48h, and 72h of the experimental time.

Clone formation assay

One thousand cells were seeded in 6-well plates and place them in a 37 °C constant temperature incubator. After 2 weeks, cells were fixed with 4% paraformaldehyde for

30 min and stained with 0.1% crystal violet for 15 minutes. The colonies were imaged and counted.

5ethynyl-2′-deoxyuridine (EdU) incorporation assay

Put 30,000 cells/well in 96-well plates with 100 μl medium with 10% FBS. After 24 h of incubation, 1 μM EdU was added to each well and incubated for 2 h at 37 °C. Cells were then permeabilized with 0.5% Triton X-100 for 10 min after fixed with 4% paraformaldehyde for 30 min at 37 °C, followed by EdU staining with Apollo dyeing reaction for 30 min. Subsequently, Hoechest was added to visualize the nuclei for 30 min, and the proportion of nucleated cells stained with EdU was observed under a fluorescence microscope.

Cell Cycle Analysis

Cell cycle was analyzed by flow cytometry, using cell cycle staining kit (MultiSciences, China). After 48h of transfection, cells were harvested and washed with PBS and then fixed in 75% ethanol overnight at -20°C. On the next day, cells were washed with PBS three times and incubated with propidium iodide (10mg/ml) for 30min at room temperature. The percentage of cells in each phase was analyzed with FACS Calibur flow cytometer, Cell Quest (BD Biosciences, USA).

Reactive oxygen species (ROS) assessment

The intracellular ROS levels were detected by Reactive Oxygen Species Assay Kit (Shanghai yeasen Technology Co.). After transfection for 48 h, DCFH-DA reactive oxygen probe was added into medium for 30 min at 37 °C under sheltered conditions. After washing with medium without FBS, ROS generation was observed under a fluorescence microscope.

Animal Experiment

We used four-week-old male BALB/c nude mice (Vital River Co., Ltd., China) for animal studies. PBS suspensions containing 1 million QBC939 cells were injected

subcutaneously into nude mice. After the tumor appeared, the tumors were measured every 3 days until these were harvested 22 days later. Tumor volume is calculated using the following formula: volume = width$^2$ × length/2.

**Table S1. Primary antibodies and secondary antibodies for Western blotting**

| Antibody | Article number | Company |
|---|---|---|
| NUF2 | ab180945 | Abcam |
| TFR1 | ab214039 | Abcam |
| p38 | ab170099 | Abcam |
| p-p38 | ab195049 | Abcam |
| JNK | ab199380 | Abcam |
| p-JNK | ab215208 | Abcam |
| ERK | ab184699 | Abcam |
| p-ERK | 4370 | Cell Signaling Technology |
| NBR1 | 20145 | Cell Signaling Technology |
| NDP52 | 60732 | Cell Signaling Technology |
| OPTN | 70928 | Cell Signaling Technology |
| TOLLIP | 4748 | Cell Signaling Technology |
| E-cadherin | 3195 | Cell Signaling Technology |
| N-cadherin | 4061 | Cell Signaling Technology |
| Snail | 3879 | Cell Signaling Technology |
| Cyclin D1 | 2922 | Cell Signaling Technology |
| Vimentin | 3932 | Cell Signaling Technology |
| P62 | P0067 | Sigma Aldrich |
| LC3 | 3868 | Cell Signaling Technology |
| β-Actin | 81115-1-RR | Proteintech |
| Anti-rabbit IgG (H+L) | 7074 | Cell Signaling Technology |
| Anti-rabbit IgG (L) | SA00001-7L | Proteintech |

S1

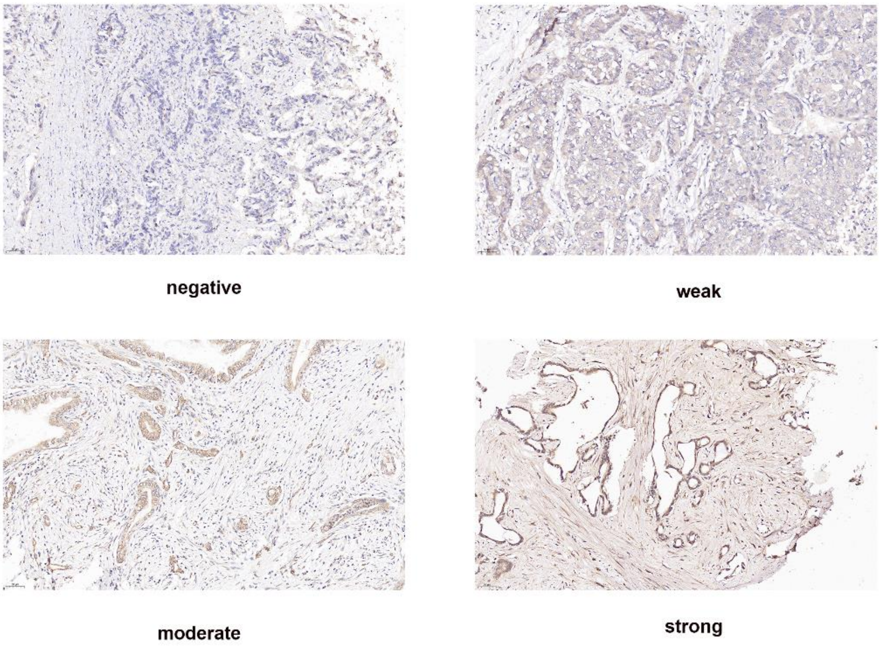


SUPPLEMENTARY FIGURE 1 Representative IHC images for the scoring criteria

S2

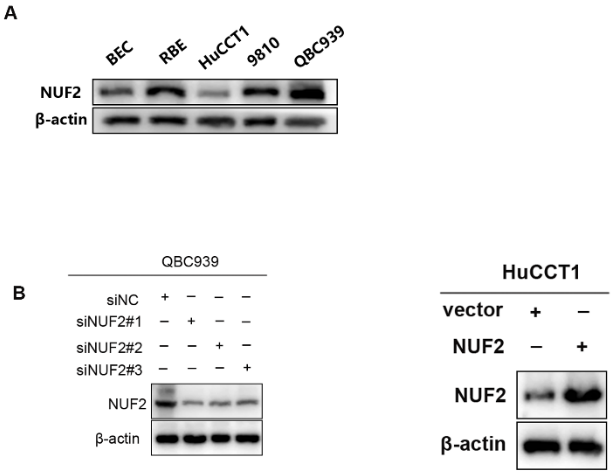


SUPPLEMENTARY FIGURE 2 Expression of NUF2. (A) Expression of NUF2 in five cell lines. (B) Validation of knockdown and over expression efficiency of NUF2.

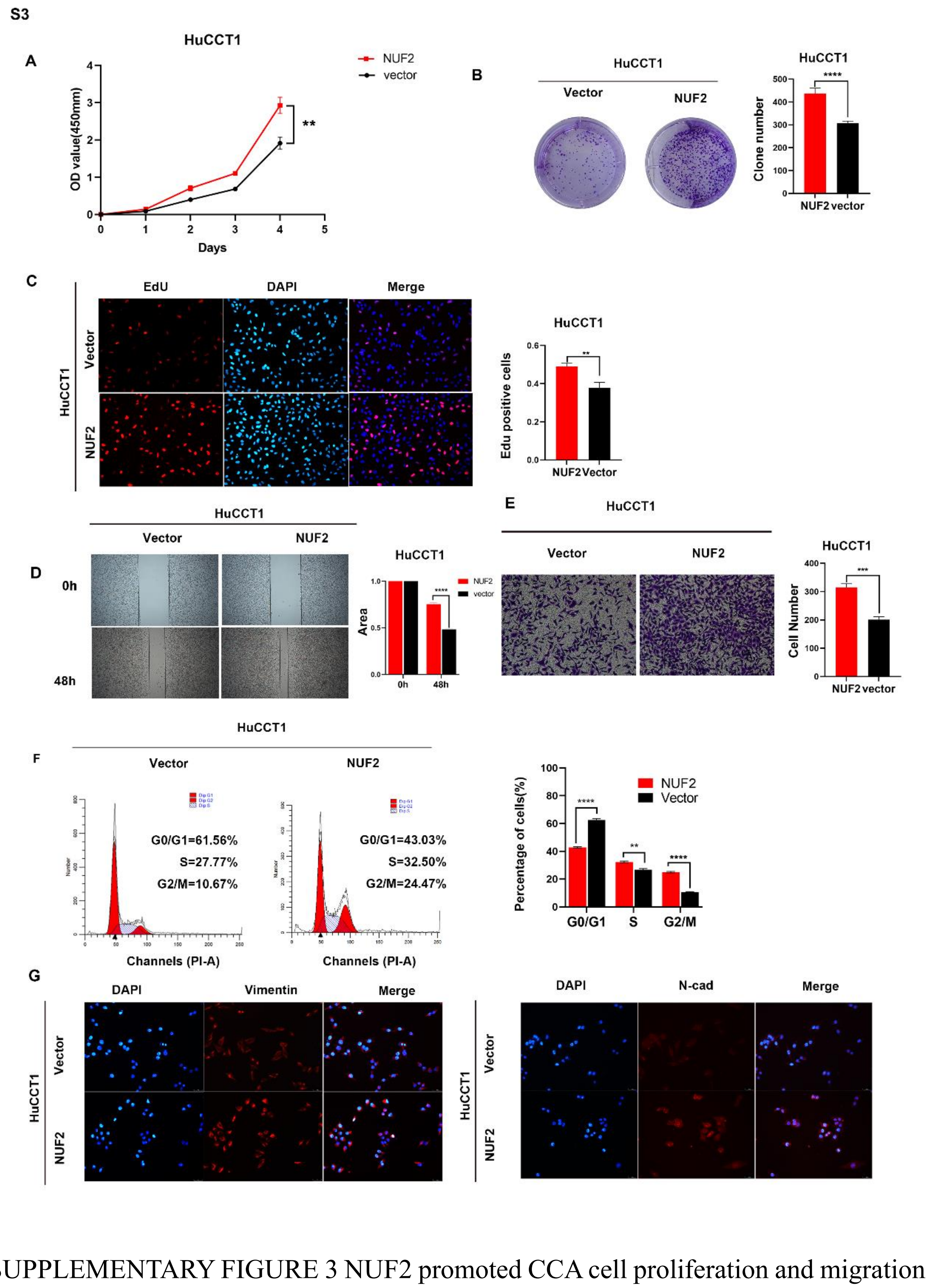


SUPPLEMENTARY FIGURE 3 NUF2 promoted CCA cell proliferation and migration in vitro. (A) CCK8 assays, (B) clone formation, (C) EdU assays were performed to identify the proliferation ability after NUF2 knockdown or overexpression. (D) Wound healing and (E) transwell assays were performed to identify the migration ability after NUF2 knockdown or overexpression. (F) Flow cytometry analysis of the cell cycle of CCA cells. (G) The expression of N-cad and Vimentin was evaluated by IF staining.

S4

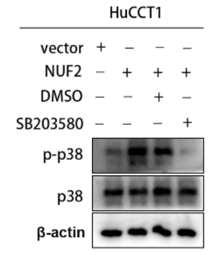


SUPPLEMENTARY FIGURE 4 Efficiency of SB203580.

S5

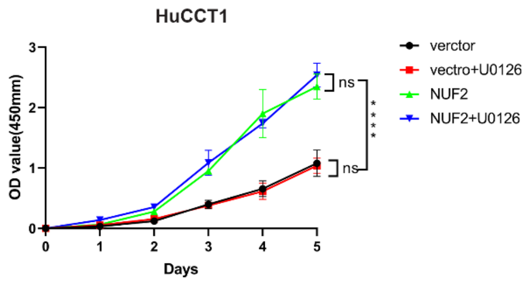


SUPPLEMENTARY FIGURE 5 Effect of U0126 on NUF2 promoting CCA.

S6

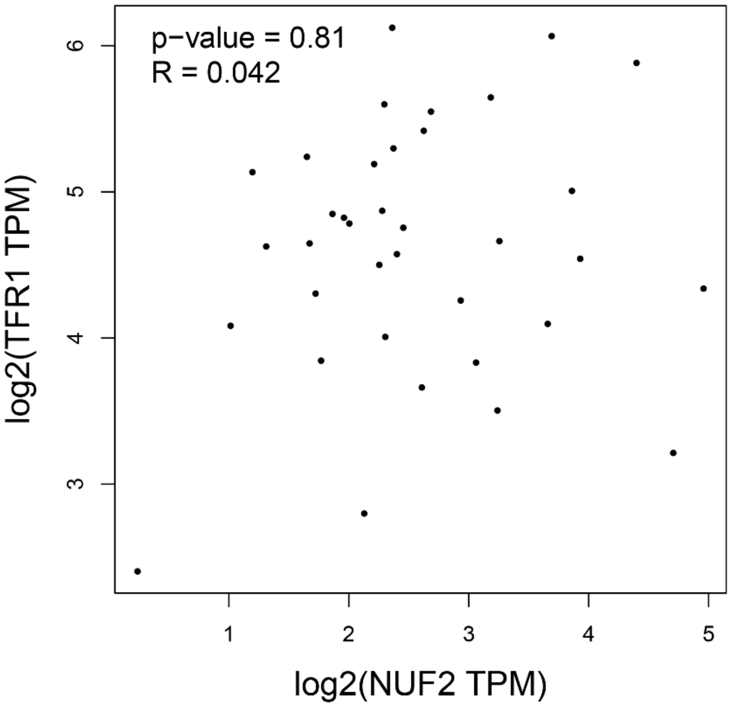


SUPPLEMENTARY FIGURE 6 Relationship of NUF2 and TFR1 in public database.

S7

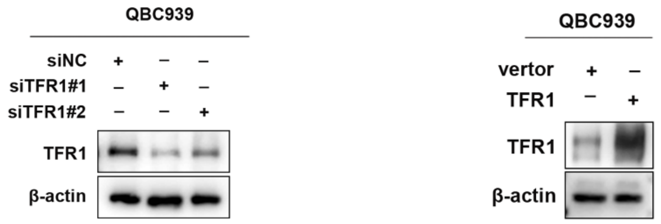


SUPPLEMENTARY FIGURE 7 Validation of knockdown and over expression efficiency of TFR1.
